# Temporary Wettability Tuning of PCL/PDMS Micro Pattern Using the Plasma Treatments

**DOI:** 10.3390/ma12040644

**Published:** 2019-02-20

**Authors:** Wei-Chih Lin, Nur Adila Mohd Razali

**Affiliations:** Department of Mechanical and Electro-mechanical Engineering, National Sun Yat-sen University, Kaohsiung 80424, Taiwan; didie.razali@gmail.com

**Keywords:** plasma treatment, surface modification, surface wettability

## Abstract

Surface wettability plays an important role in determining the function of a wound dressing. Dressings with hydrophobic surfaces are suitable for bacterial adsorption, however, a hydrophilic surface is needed to improve cell attachment for most anchorage-dependent cell types. Furthermore, the hydrophobicity/hydrophilicity of the surface can be used to direct cellular processes such as cell initial attachment, adhesion, and migration during wound healing. Thus, a surface with an ability to switch their surface wettability improves the practicality of the dressing. In this study, we propose a temporary surface wettability tuning for surface patterning utilizing plasma treatment. Polycaprolactone (PCL) and polydimethylsiloxane (PDMS) surfaces were treated with tetrafluoromethane (CF_4_), sulphur hexafluoride (SF_6_), and oxygen (O_2_) plasma, and the effects on the surface wettability, roughness, and chemical composition were investigated. Based on the contact angle measurement, CF_4_ plasma altered surface wettability of PCL and PDMS films to hydrophobic and hydrophilic, respectively. After CF_4_ treatment, better attachment of primary mouse embryonic fibroblast cell (3T3) was observed on the treated PDMS surface. Embedding PCL into PDMS generated a hydrophobic-hydrophilic pattern mixture surface, which offers great potential in the tissue engineering field such as cell patterning and guidance.

## 1. Introduction

Conventionally, an infected wound is treated using antimicrobial agents [1,2] such as silver nanoparticles and bacterial cellulose. Recently, several articles have been issued to demonstrate the application of hydrophobic surfaces for bacterial removal in a wound dressing. Dressing surface coated with dialkyl carbomyl chloride (DACC) increase the hydrophobic characteristic for strong bacteria-reducing effects [3]. The relationship between hydrophobicity and infection are discussed in Reference [4]. However, the hydrophobic surface can reduce the adherence of the dressing to the skin, while hydrophilic surfaces are proven to have better adherent capabilities. Thus, temporary wettability tuning from hydrophobic to hydrophilic improves the properties of a material for wound dressing application. The ability of a hydrophobic surface to tune to hydrophilic after bacterial absorption helps secure the dressing in position and improve its practicality. A hydrophilic surface is preferred for most attachment and adhesion of anchorage-dependent cell types during the wound healing process. Temporary surface wettability tuning can be achieved by chemical coating, plasma modification [5,6,7], ultraviolet irradiation, and corona discharge. Due to simple, low cost, and large coverage area, plasma treatment has received great attention for surface modification in academic and industry fields. Incorporating chemical groups during the interaction of plasma particles with material surface results in changes in material energy [8,9,10], functionality, wettability, and morphology within a short period [11] of treatment time.

Biomedical, implantable drug delivery systems made from glass, silicone elastomer, or plastic materials permanently remain in the biological tissue if not surgically removed. Due to the inherent difficulty associated with retrieving small-scale devices from tissues, it is advantageous to apply biodegradable polymers, where the micro devices would naturally degrade in tissues over a desired period of time. Thus, polycaprolactone (PCL) is widely used as a drug delivery carrier [12] and artificial scaffolds [13] due to its biodegradability. Furthermore, beneficial characteristics such as inert, transparent at optical frequency, and biocompatible make polydimethylsiloxane (PDMS) the most extensively used material for lab-on-chip and biosensors. However, polymeric biomaterials are often surface-modified [14,15,16] to meet the demand requirements in biomedical and tissue engineering. Modifications of PCL and PDMS surfaces are necessary to increase their wettability [17,18,19], and provide a friendly bio-environment for cell culture. With regard to cell attachment issues, PCL nanofibers have been modified using low-pressure radio frequency (RF) discharge plasma treatment and the results show an improvement in cell attachment [18,20].

The effects of surface alteration on plasma treatments depend on treatment parameters such as applied power, pressure, and time. For instance, the effect of applied pressure on the fluorination of treated polymer has been previously discussed [21]. When the pressure is applied up to a threshold, the treated surface is saturated with fluorine due to the full dissociation of the gas molecules in plasma. In contrast, pressure above the threshold results in a significant decrease of fluorine while increasing oxygen concentration, thus forming a hydrophilic surface. Another study states that adjusting CF_4_ plasma power and treatment time enables a switchable surface wettability [22]. Based on the result, the treated polymer surface turned from hydrophobic to hydrophilic under relatively low applied power. Furthermore, plasma treatment coupled with masking techniques allows different chemical and/or topographical areas on the same substrate [15]. This simple approach for selective modification allows cell patterning, and is used to direct cellular processes such as cell migration, differentiation, and adhesion [23,24,25]. A stepwise gradient wrinkle pattern with spatially-controlled wavelengths can be achieved based on sequential oxygen plasma treatments. A research team [26] fabricated the wrinkle pattern in three uniform region sizes, and showed that the wettability of the wrinkle pattern is dependent on wrinkle features.

This research proposed a temporary wettability tuning method of PCL and PDMS surfaces using the plasma treatment method. Modification via plasma treatment gives a temporary, non-permanent modification effect on the surface, as the treated surfaces recover to their initial surface characteristics. The effect of plasma treatment on wettability tuning, which was separately modified by oxygen (O_2_), tetrafluoromethane (CF_4_), and sulphur hexafluoride (SF_6_) was experimentally investigated. The effects of applied power and treatment time on the final surface wettability of polymers were investigated. We then cultured mouse 3T3 fibroblast cells on the treated surface to demonstrate the possibility and reliability of surface wettability control using a plasma treatment method.

## 2. Materials and Methods

### 2.1. Fabrication of PCL and PDMS Film

PCL are biocompatible, thermally induced shape memory polymers, which have been intensively used to control cellular interactions in biological and tissue engineering [27,28] applications. PCL melting temperature (T_m_) changes the mobility of polymer chains [29], hence, allowing for shape memory effect. However, tuning the T_m_ is needed as it is relatively high around 60 °C. To improve the suitability of PCL in tissue engineering, T_m_ was modulated, making it actuate near body temperature, which can be achieved by controlling the nano-architecture of cross-linked PCL. In this research, two-branched (2b20) and four-branched (4b10) PCL macro monomers were synthesized using ring-opening polymerization of caprolactone from tetramethylene glycol hydroxyl end-groups [30]. The mixture ratio of 2b20 and 4b10 (2b20:4b10) PCL macro monomers was applied to synthesis 1:1 (50%:50%), and 7:3 (70%:30%) PCL films to program the melting temperature at 33 °C and 38 °C, respectively. Furthermore, the mixture of macro monomers was dissolved in 45% concentration level of xylene (Sigma Aldrich, St. Louis, MO, USA). Subsequently, 10 wt.% of benzoyl peroxide (BPO, Sigma Aldrich, St. Louis, MO, USA) initiator was added for the cross-linking process. Two glass slides with a 300 μm spacer were utilized to control the fabricated PCL thickness. The solution was filled into the space gap and placed in a preheated oven at 60 °C for 12 h.

PDMS elastomers (Dow Corning’s Sylgard® 184, Midland, MI, USA) were used to fabricate the PDMS film. The PDMS resin was mixed with the curing agent at 10:1 weight ratio and the pre-polymer mixture was thoroughly mixed to ensure that the curing agent is evenly distributed for proper polymerization. The pre-polymer mixture was then poured into the space-controlled gap, formed by two polystyrene slices with a spacer. The mixture was then placed in a vacuum desiccator for 30 min to eliminate air bubbles formed during the mixing process. Lastly, the mixture was cured in the preheated oven at 60 °C for 12 h and the fabricated film was removed from the spacer.

Then, the PCL/PDMS microstructure was fabricated using the conventional soft lithography method. Firstly, the PDMS pre-polymer mixture was poured onto a master mould to obtain a square microchannel structure. After curing, the PDMS was removed from the mould, and the imprinted microchannel PDMS film was sealed to a glass slide. The PCL (1:1) solution was injected into the channels, and the sample was cured again in a preheated oven at 60 °C for another 3 h. The sample was removed from the glass slide and a Scanning Electron Microscope (SEM, JEOL JSM630, Tokyo, Japan) was used to observe the PCL/PDMS microstructure.

### 2.2. Plasma Treatment

**Figure 1:**
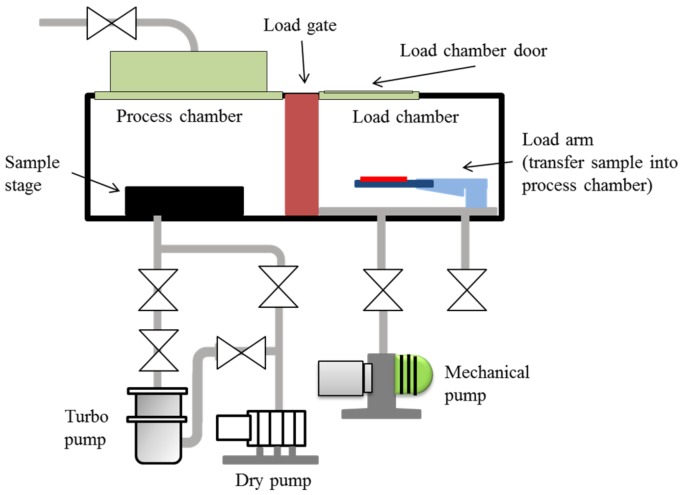
Schematic diagram of the plasma system for sample treatment.

Plasma treatment, the most efficient way to modify surfaces uses ionized gas to functionalize the outer space [31]. In this study, we used an inductively coupled plasma system (ICP AST Cirie-200, Advanced Technology System, Hsinchu, Taiwan) to introduce oxygen and fluorinated plasmas into the chamber. Radio frequency (RF) power was inductively coupled via the copper coil to create the plasma. The plasma was generated at 13.6 MHz RF electromagnetic field, at 100 mTorr gas pressure and 60 W discharge power. The whole process was conducted in an automatic mode involving: (1) venting the load chamber, (2) setting the sample on the load lock arm, (3) loading the sample into the process chamber, (4) etching process of the sample, and (5) the unloading of the sample back to the load chamber. The chamber was separated into load and process chambers by the load gate. Initially, the base pressure in the process chamber was kept at 5 × 10^−5^ Torr. Then, the load chamber was vented to 7.6 × 10^2^ Torr (approximately 1 min) to open the load door. The sample was aligned in the load arm, and the load chamber was evacuated. When the crossover pressure was reached, the sample was transferred into the process chamber. The sample was placed on the sample stage, the load gate valve was closed, and the chamber was evacuated to the base pressure (5 × 10^−5^ Torr) again. After the base pressure was reached, the plasma was introduced into the chamber at 30 sccm flow rate. The etching process was performed at 2 mTorr, after which the sample was automatically unloaded back to the load chamber, and the chamber was vented to the atmosphere. The schematic diagram of the plasma system is shown in Figure 1.

Key parameters of the plasma process are the RF power and treatment time, which determine the plasma intensity and applied plasma dosage, respectively. In this study, the RF power was varied at 30 W and 60 W while the treatment time was varied from 10 s to 180 s, to study the effect of these parameters on the treated surface sample. Table 1 shows the samples abbreviation based on the macro monomer composition and the applied RF power.

### 2.3. Characterization of Plasma Treated Surfaces

Plasma treatment alters the surface characteristics of polymeric biomaterial such as surface roughness, wettability, surface energy, and biocompatibility. Etching, free radical forming, and ion bombardment reactions on the treated surface are the three reactions that occur during plasma treatment. Surface roughness and morphology change, respond to the etching reaction and treatment conditions such as treatment time, plasma power, and gas flow rate [5]. Characterizations of the treated surface were carried out using atomic force microscope (AFM), contact angle (CA) measurement, and X-Ray Photoelectron spectroscopy (XPS) analyses.

#### 2.3.1. Surface Roughness

Change in surface morphology, branched polymer chain, and atoms implantation occurred simultaneously on the surface of the polymer when the polymer was exposed to the plasma. Surface roughness was highly dependent on the treatment parameter in response to the etching reaction. Plasma modified and unmodified PCL and PDMS films were characterized in terms of surface roughness using atomic force microscope (AFM, SPM-9500, Shimadzu, Kyoto, Japan) to quantify the roughness variation on the micro and nanoscale. An AFM probe was used to inspect free radical forming and ion bombardment reactions on the surface of treated films caused by plasma etching reactions. The measurement was operated in tapping mode using AFM tip (RFESP-75, Veeco, Plainview, NY, USA) with a nominal spring constant of 3 N/min and approximate resonant frequency around 75 kHz in the ambient environment. The scanning area for image comparison was kept at 25.0 μm^2^ with a scanning rate of 0.5 Hz. The measurements were replicated five times and the data were picked up randomly at the surface of the sample.

#### 2.3.2. Wettability

The relationship between surface roughness and wettability stated that due to the chemistry on the surface, the surface roughness will enhance wettability. Wettability, which refers to the hydrophobicity/hydrophilicity of the surface, is one of the most apparent results of plasma treatment. Surface wettability affects the biological response to the implanted material such as protein adsorption and cell attachment. Thus, it is important to characterize the effect of plasma treatment on the wettability of treated polymer surfaces. Wettability studies engage with contact angle measurements as the primary data, which indicate the degree of wetting when solid and liquid objects interact [32]. Contact angle measurements were applied to measure solid surface wettability where the angle showed a liquid contact with three-phases of matter including solid, liquid, and gas intersections. A contact angle measurement system (DropMaster DM-701, Kyowa, Japan) with the sessile drop method was applied, and the wettability was analyzed using the built-in interface measurement and analysis FAMAS Basic software. Static mode on leveled surfaces was set, and the measured contact angle, which is in accordance with the ideal contact angle, was calculated by the well-known Young equation to quantify surface energy.

#### 2.3.3. X-Ray Photoelectron Spectroscopy 

XPS analysis was performed to determine the branching polymer chain and implanting atom upon plasma treatment. The elemental composition and chemical state on the plasma treated films’ surface was measured by X-ray photoelectron spectroscopy (XPS, PHI-5400 ESCA Electron Spectrometer, PerkinElmer, Waltham, MA, USA) equipped with a monochromatic Al Kα X-ray source and a spherical section analyzer. A typical depth of modification is approximately 5–50 nm, which can be measured precisely using XPS. The X-ray beam power was set to 100 W and raster scan was over 1.4 mm by 0.3 mm area on the samples. Elemental high-resolution scans for C1s were referenced to the 1s aliphatic carbon at 285 eV. The take-off angle between the sample’s surface and electron optical axis were collected at 45° for survey and high-resolution spectra. Survey spectra were collected with pass energy at 280 eV in 0.5 eV step (wide), and 55 eV in 0.1 eV step (narrow).

### 2.4. The Attachment of 3T3 Fibroblast Cells

Numerous studies claim that surface biocompatibility in terms of protein adsorption and cell attachment change upon plasma treatment [33,34]. Protein adsorption to surfaces is a dominant factor for determining cellular interactions with the surface of the biomaterial used. The adsorbed protein amounts are influenced by the hydrophilicity/hydrophobicity characteristics of a surface where the amount of absorption is positively correlated with surface wettability alteration. This study was conducted to further confirm the surface wettability alteration on PCL and PDMS after plasma treatment. In this study, the protein used was bovine serum albumin labeled with fluorescein isothiocyanate (FITC-BSA). BSA is known as a coating adhesive protein, which binds to the hydrophilic charge domain, in contrast to the hydrophobic charge domain—which is incompatible for the binding.

A protein adsorption study was conducted following the procedure from [35]. Immediately after CF_4_ plasma treatment, PCL and PDMS samples were incubated in FITC-BSA with 100 µg/mL of BSA solution in PBS. The samples were treated in a dark room for 2 h to allow the adsorption process. Throughout the adsorption process, a magnetic stirrer was used to avoid salt precipitation. After 2 h, the excess solution was removed and the samples were washed with deionized (DI) water. Thorough rinsing with DI water was performed to remove any residual protein. Subsequently, the samples were dried in a nitrogen atmosphere and stored in a desiccator overnight, prior to analysis. Fluorescence microscopy was performed at 450 nm excitation and the fluorescence images were analyzed using the software ImageJ 1.52a.

In addition, surface energy, surface roughness, or chemical composition affect the interaction and attachment of cells with biomaterials [15,36]. For this reason, our study helps improve the understanding of how material surface properties influence cellular responses at the material interface, so as to develop general principles that can be used to engineer clinically useful implantable devices and tissue-engineered constructions. The primary 3T3 fibroblast cells were cultured on the plasma treated samples and the cell’s initial attachment was observed. 3T3 fibroblast cells were cultured at a density of 1.0 × 10^4^ cell/cm^2^ on PCL and PDMS films in 12-well plates for 24 h. The cells were maintained in Dulbecco’s Modified Eagle’s medium (DMEM, Gibco, Carlsbad, CA, USA) supplemented with 1% penicillin/streptomycin with 10% fetal bovine serum (FBS, PAA Laboratories, Pasching, Australia), and incubated in a humidified atmosphere containing 5% CO_2_ at 37 °C under standard condition. Phosphate buffered saline (PBS, Sigma Aldrich, St. Louis, MO, USA) was then used to wash the cells, and the cells were fixed for 30 min with freshly prepared 4% paraformaldehyde in PBS. The fixed cells were washed for the second time using PBS followed by a permeabilization treatment. After 5 min of permeabilization using blocking buffer (0.1% Triton X-100, 1% BSA in PBS), the cells were incubated with rhodamine phalloidin diluted at 1:100 in PBS for 15 min. Thereafter, the PCL and PDMS samples were washed with PBS 3 times and mounted in a mounting solution with 4’,6-diamidino-2-phenylindole (DAPI, Vector Laboratories, CA, USA) for microscopy. DAPI was used to counterstain the nucleus. Cell staining was applied to facilitate the visualization and inspection of cells using fluorescence microscopy (Nikon Eclipse E400, Nikon, Tokyo, Japan).

## 3. Results

### 3.1. PCL/PDMS Microstructure

**Figure 2:**
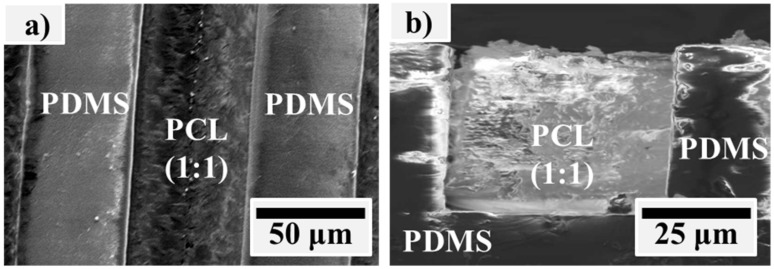
SEM images of the fabricated PCL/PDMS micro structure from the (**a**) top view and (**b**) side view.

PCL solution was embedded into PDMS with a square micro channel to form the PCL/PDMS micro structures. Figure 2 shows the SEM image of the fabricated PCL/PDMS micro structure sample. This micro pattern co-surface achieved two opposite wettabilities on one surface. It also delivered topographic signal and provided cells with precise instructions to properly control cellular processes such as attachment, adhesion, migration, and differentiation.

### 3.2. Surface Roughness

**Figure 3:**
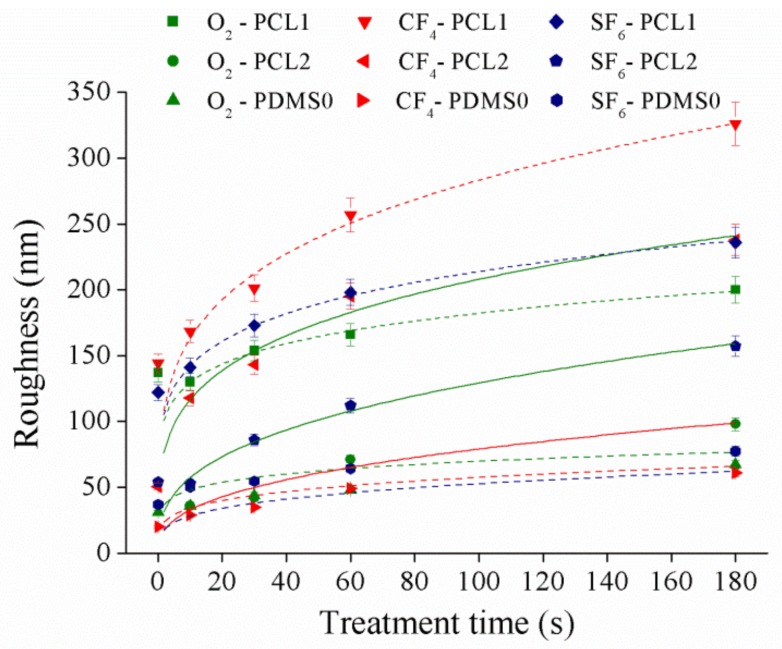
The surface roughness of PCL and PDMS films at 30 W.

This section examines the surface roughness of the samples as it may greatly alter their surface morphology after plasma treatment. Surface roughness of the films at 30 W was measured by AFM, and the result is presented in Figure 3. The data presented were the average of five replicated measurements where the data were randomly measured from three samples, and roughness observations were quantified in terms of root mean square values.

According to the obtained data, the roughness for both PCL and PDMS surfaces increased with respect to plasma treatment time. PCL samples showed the highest roughness increment upon CF_4_ plasma treatment compared to O_2_ and SF_6_ plasma, while the PDMS sample showed the most significant increase in roughness after the SF_6_ plasma treatment. After CF_4_ plasma treatment, the PCL1 roughness increased ~181.9 nm, PCL2 increased ~187.3 nm, while PDMS0 roughness increase ~41.1 nm. The change in surface topography after plasma treatment is explained when the highly unstable species in the plasma gas collide with some polymer chains in the topmost layer [37]. Collision phenomena promote chain scission leading to low molecular weight oligomers formation, which can be removed from the surface. Material removal changed surface topography and roughness. According to [38], surface roughness increased significantly when the applied power was less than 70 W, while a weak roughness appeared on higher applied power. When the applied power was low (30 W and 60 W), the etching process had a more noticeable effect on the treated surface [38,39]. Thus, a significant surface roughness increase was observed. Our results show a similar trend to a study conducted in [37]. In [37], a significant increase in surface roughness is obtained where the roughness of polyethylene surface increased ~123.6 nm (percentage increase of 464.6%) after atmospheric plasma treatment compared to the untreated surface.

### 3.3. Surface Wettability

**Figure 4:**
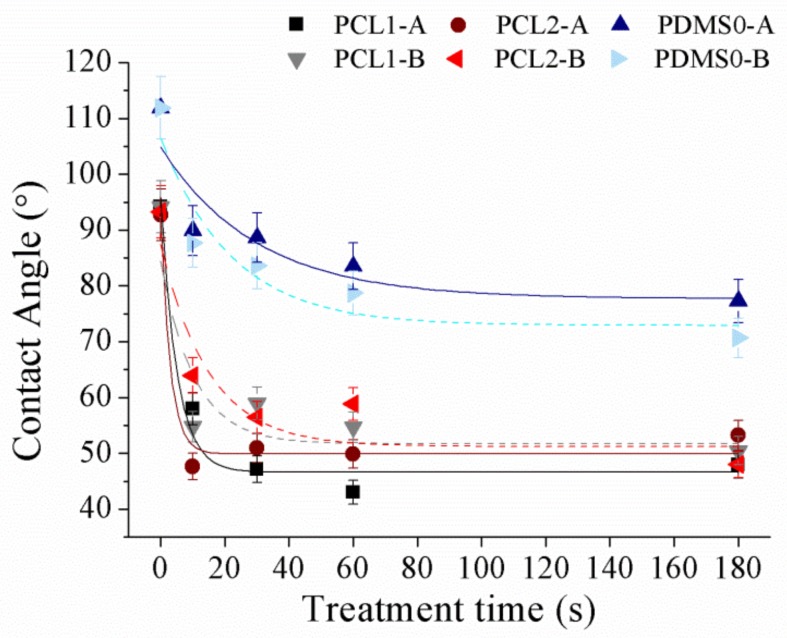
The water contact angle of PCL and PDMS films after being treated with O_2_ at different parameter settings.

Surface wettability of PCL and PDMS surfaces was measured using sessile drop analysis. Hydrophilic surfaces with small contact angles (less than 90°) corresponded to high wettability, while hydrophobic surfaces with large contact angles (more than 90°) corresponded to low wettability. PCL and PDMS surfaces were modified separately by applying various plasma powers (30 W and 60 W) at 10, 30, 60, and 180 s in an oxidized and fluorinated environment. Figure 4 demonstrates the contact angle of polymer changes after the O_2_ plasma process.

After the O_2_ plasma treatment process, we observed that surface hydrophilicity of PCL and PDMS surfaces was increased. The measured contact angle decreased with the increase in treatment time. For the treated PCL1 film, the contact angle decreased from 94.2° to 47.9° at 30 W and from 94.1° to 50.4° at 60 W. Meanwhile, PCL2 decreased from 92.7° to 53.2° and from 93.3° to 48° at 30 W and 60 W, respectively. The contact angle of the PDMS0 film surface decreased by 41.7°, from 119° to 77.3° at 30 W and decreased by 48.3° which was from 111.9° to 70.7°. The increase in surface wettability after O_2_ plasma treatment was due to the introduction of hydrophilic groups [40,41,42].

Oxygen-containing functional groups were generated on the treated surface after treatment, and the chemical change in the upmost surface layer transformed hydrophobic surfaces into hydrophilic ones. For example, PDMS surface change from silane (Si–CH_3_) to silanol (Si–OH) after O_2_ plasma treatment, which increases its hydrophilicity. Although both PCL and PDMS showed increased wettability, they showed a moderately high contact angle after treatment. The measured contact angles corresponded to the obtained AFM result, where the surface roughness of PCL and PDMS surfaces increased significantly after O_2_ treatment. As surface roughness is the key factor for the wettability, this high surface roughness induces hydrophobic [43,44] surfaces as a known example of the lotus effect. Surface roughness reduces surface energy and might consequently reduce the wettability of molecular liquids. Hence moderate high contact angles after treatment were measured.

**Figure 5:**
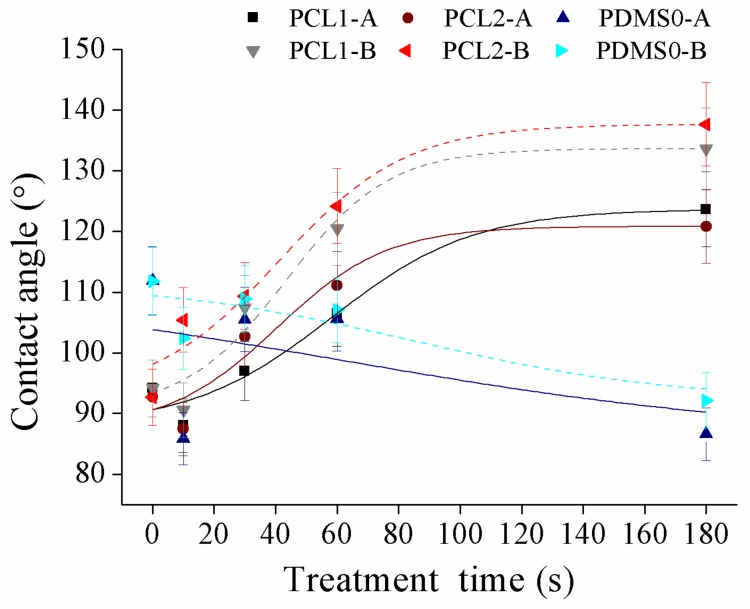
The water contact angle of PCL and PDMS films after treatment with CF_4_ at different parameter settings.

Then, the analysis was continued by studying CF_4_ plasma treatment effects on surface wettability and the result is presented in Figure 5.

Figure 5 shows the contact angle after CF_4_ treatment of PCL1, PCL2, and PDMS0 films at different parameter settings. Compared to the increase in surface wettability of both PCL and PDMS after O_2_ plasma treatment, the surface wettability of CF_4_ treatment showed an opposite characteristic. According to the result, the wettability of PCL surfaces decreased while that of PDMS increased. The contact angle of PCL1 increased from 94.2° to 123.7° with 30 W and from 94.2° to 133.7° with 60 W, and contact angle for PCL2 increased from 92.7° to 120.8° (30 W) and from 92.7° to 137.7° (60 W). In contrast, the PDMS0 surface increased its hydrophilicity as the contact angle decreased from 119° to 86.6° (30 W) and from 119° to 92.15° (60 W). Since the roughness of PCL and PDMS surfaces increased after CF_4_ plasma treatment, the measured PDMS contact angle after CF_4_ plasma treatment was contrary to the expectation. The decrease in contact angle indicates that the PDMS surface loses its hydrophobicity compared to the untreated surface. The study in Reference [45] states that the introduction of fluorine during plasma treatment may be overcompensated by the consequence of oxidation effects associated with the plasma treatment. Chemical re-orientation occurs when fluorine—that is initially on the surface—re-orients towards the bulk, which exposes oxygen to the surface. This will result in increased hydrophilicity of the PDMS surface after fluorine introduction.

**Figure 6:**
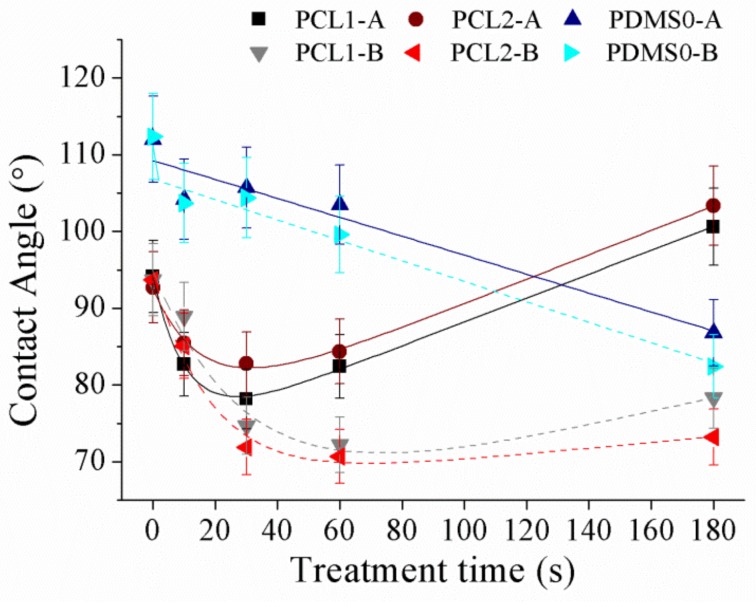
The water contact angle of PCL and PDMS films after treatment with SF_6_ at various parameter settings.

Next, the effects of SF_6_ treatment on PCL and PDMS surfaces were investigated. A similar trend as CF_4_ on surface wettability was achieved as shown in Figure 6.

The obtained result show that the PCL contact angle increased with 30 W applied power which was from 94.2° to 100.6° and 92.7° to 103.4° for PCL1 and PCL2, respectively. However, at 60 W, both PCL1 and PCL2 films showed an increment in wettability as the contact angle decreased from 93.7° to 78.3° and from 93.7° to 73.2°. The result shows that PCL films increased its hydrophobicity at 30 W, while increasing its hydrophilicity at 60 W. Figure 6 shows that the water contact angle of PCL surfaces decreased when the samples were first exposed to SF_6_ plasma (10 and 30 s). Prolonging treatment time to 60 and 180 s increased the water contact angle of PCL surfaces. Treatment time influenced the grafting and etching balance during plasma treatment, where the etching and grafting processes synergistically affected the final wettability of the fluorinated surface [22]. The initial decrease in contact angle within short treatment time is possibly caused by the plasma etching and oxidation for surface cleaning before the activated sites had enough time to react with fluorine [46]. Thus surface cleaning and oxidation based on plasma etching alters the surface topography of polymers and their wettability without modifying their surface texture. Within this time, surface roughness was greatly enhanced and increased the surface’s hydrophilicity. Meanwhile, the grafting effect became major for surface wettability gradually with treatment time. Longer treatment time resulted in significantly increased fluorine amounts on the PCL surface, whereas the oxygen concentration decreased, leading to the formation of the hydrophobic surface. For PDMS, the film increased its hydrophilicity after SF_6_ treatment regardless of the applied power. The contact angle decreased from 112° to 86.8° (30 W) and from 112.4° to 82.4° (60 W).

Furthermore, XPS measurements were conducted in order to acquire more details on the surface functional groups, induced by plasma treatment.

### 3.4. Surface Composition

XPS measurements were done to get a deeper insight into the surface wettability modification by quantifying the changes in surface chemistry, produced by plasma treatment.

Elemental composition on the surface of PCL1, PCL2, and PDMS0 treated by CF_4_ at 30 W at different treatment times are summarized in Table 2. The atomic compositions for carbon and oxygen were altered upon plasma treatment compared to the untreated surface, for all samples. After CF_4_ treatment, fluorine appeared on the surface of all samples as expected. The surface fluorine content increased approximately 15 at. % to 20 at. % after 10 s of fluorination on PCL and there was no significant increment detected until 180 s. However, fluorine content was only observed after the 30 s treatment time and increased approximately to 10 at. % after 180 s. Based on the contact angle measurement done after CF_4_ plasma treatment (Figure 5), the contact angle of PDMS surface after being treated with CF_4_ plasma for 10 s was slightly lower than that of the PCL surface. The lower contact angle of PDMS indicates that the surface energy of PDMS is higher than PCL. As the surface energy increases, it is more difficult for the F element to bind to the surface. Thus, more time is required for the element to bind to the surface, which may explain the absence of F elements on the PDMS surface after 10 s. The low amount of fluorine suggests that the presence of fluorine atoms is merely on the upper level of the PDMS treated surface.

Table 3 shows the atomic composition of PCL and PDMS surfaces after SF_6_ plasma treatment. After fluorination, the atomic concentration of fluorine increased significantly on the PCL surface in response to treatment time compared to the PDMS surface. Furthermore, introducing fluorine elements reduced the Si composition on the surface.

**Figure 7:**
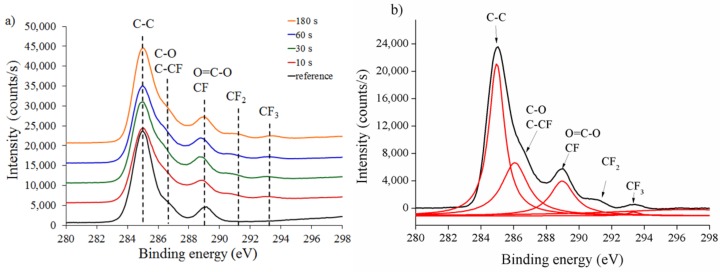
PCL1 (**a**) XPS survey spectra at various times and (**b**) peak deconvolution at 180 s. The “reference” represents the untreated (0 s treatment) PCL1 sample.

We then recorded high-resolution C1s spectra to further characterize the chemical structure of untreated and treated PCL and PDMS surfaces using CF_4_ plasma treatment. Figure 7a presents the comparison of XPS C1s spectra on PCL1 surface at different treatment times.

CF_4_ treatment forms a fluorine-containing group coating (CF_2_ and CF_3_) on the treated surface compared to the untreated PCL surface. A peak deconvolution of the C1s procedure on the treated PCL1 surface at 180 s was carried out to evaluate C1s spectra. The detailed deconvolution curve of C1s spectra with the assigned peak is presented in Figure 7b. Five components were fitted in the C1s peak assigned to C–C at 285 eV, C–O and C–CF at 286 eV, O=C–O and CF at 288 eV, CF_2_ at 291 eV, and CF_3_ at 293 eV binding energies. The deconvolution model was assumed to be adequate, agreeing with previous studies using complex fluorinated polymer systems, after plasma treatment [47,48].

### 3.5. Fibroblast (3T3) Cell Attachment

**Figure 8:**
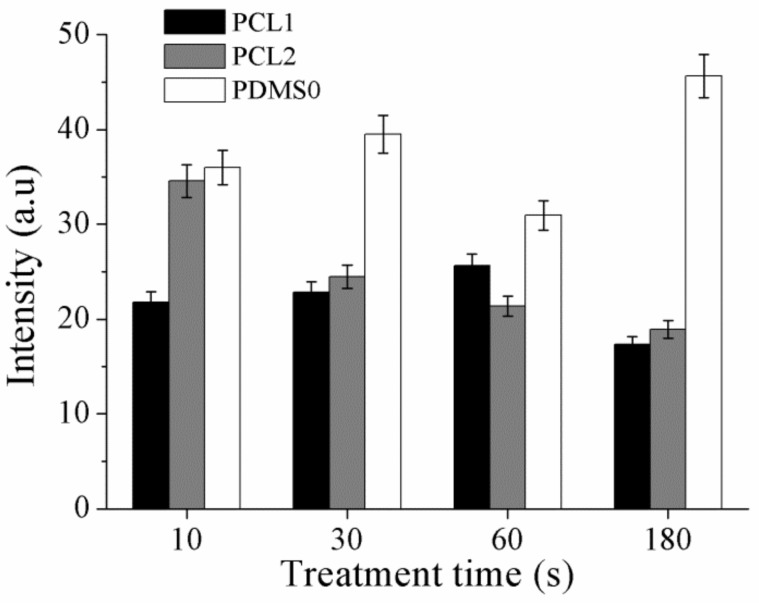
The fluorescence intensity of FITC-bovine serum albumin (FITC-BSA) adsorbed on PCL and PDMS after CF_4_ treatment.

A FITC-BSA protein was used to study surface wettability of the PCL- and PDMS-treated surfaces, corresponding to the measured contact angle and XPS result. Adsorption was determined based on fluorescence intensity where the intensity is proportional to the density of FITC-BSA on the surface [49]. Thus, the fluorescence intensity can be utilized to indicate protein adsorption on the surface, calculated using ImageJ software. The bar graph in Figure 8 shows the relative fluorescence intensity of PCL and PDMS samples normalized to the intensity of FITC adsorbed on untreated PCL samples (100%) for systematic comparison. After CF_4_ plasma treatment, the absorbed amount of BSA protein decreased on PCL1 and PCL2. In contrast, the amount of absorbed BSA protein increased on the treated PDMS surface.

The measured fluorescence intensity shows that the absorption amount of BSA protein is higher on the treated PDMS surface. Higher intensity, which indicates a higher density of FITC-BSA on the PDMS surface, is due to the high wettability of the surface. As aforementioned, BSA protein has a stronger ionic interaction on hydrophilic surfaces, thus increases the absorption amount of BSA protein. Moreover, as CF_4_ plasma treatment increased the hydrophilicity of the PDMS surface as a function of treatment time, a higher intensity of fluorescence is observed when the treatment time is increased. It is assumed the adsorbed proteins bond to the surface and form an interlayer between the surface and the cell, which influence cell attachment and adhesion.

**Figure 9:**
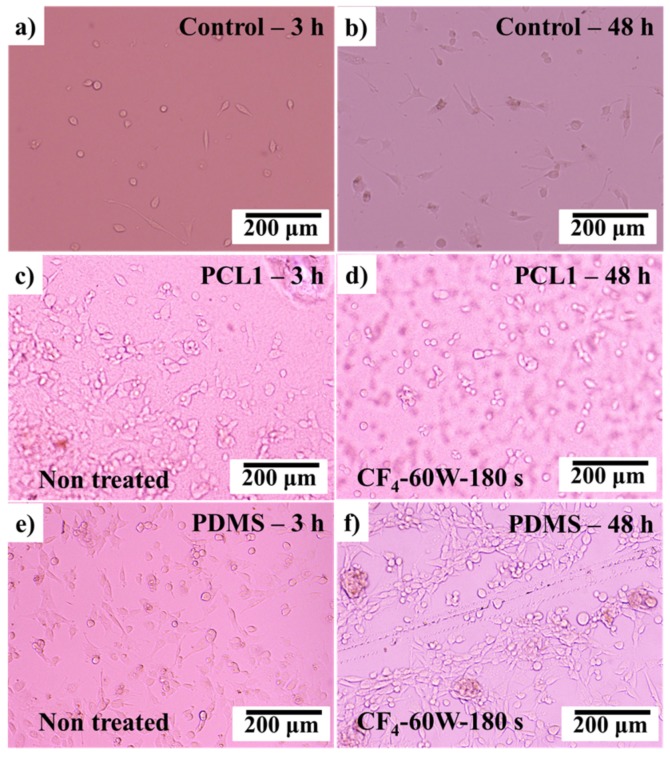
After 3 and 48 h of culturing fibroblast (3T3) cells on (**a**,**b**) control surface, (**c**,**d**) PCL1 surface treated with CF_4_, and (**e**,**f**) PDMS surface treated with CF_4_.

3T3 fibroblast cells were then cultured on the CF_4_ plasma-treated PCL and PDMS surfaces. The observations were made in the first 48 h after the culturing process, as the surface modification by plasma treatments had a major influence at the initial cell attachment process. Figure 9 shows the cultured fibroblast cells after 3 and 48 h.

As discussed in Section 3.3, after CF_4_ plasma treatment, the PCL1 film surface became hydrophobic meanwhile PDMS film surfaces became hydrophilic. By comparing the cultured 3T3 fibroblast cells on the treated PCL1 (hydrophobic) and PDMS0 (hydrophilic) surfaces, PDMS0 performed better cell attachment after 48 h of culturing time compared to PCL1. PDMS0 with a high hydrophilic surface and higher BSA molecule absorption interaction strength was compatible with prolonged cell attachment and adhesion. Compared to the PCL1 surface, this result proves that the treated PDMS0 surface influenced the initial attachment and adhesion of cultured cells.

## 4. Conclusions

In this study, we investigated a simple approach for surface wettability tuning using a plasma treatment method. According to the AFM result, a significant increase in surface roughness is obtained on PCL and PDMS surfaces after plasma treatment contributes to the low applied power. The introduction of oxygen-containing functional groups after O_2_ plasma treatment increased surface wettability of PCL and PDMS. However, hydrophobicity of the PDMS surface is reduced after fluorination using CF_4_ and SF_6_ plasma. After fluorination, the re-orientation of fluorine groups from the surface towards the bulks exposes oxygen groups, thus, increasing PDMS surface wettability. Moreover, the treatment time is a crucial factor in determining the final wettability of the treated surface. Within a short period of treatment time, the PCL contact angle decreases, while extending treatment time increases the measured contact angle again. The initial decrease in contact angle is due to surface cleaning during plasma etching, and the grafting effect becomes major for surface wettability gradually with treatment time. XPS measurement shows that the concentration of the fluorine element on the treated surface increases after fluorination. Furthermore, cultured 3T3 fibroblast cells adhered better to the PDMS surface compared to PCL, which corresponds to their surface wettability after CF_4_ treatment. Thus, by embedding PCL into the PDMS structure, a hydrophobic–hydrophilic pattern surface is achieved by CF_4_ plasma treatment, which is expected to have a great potential in tissue engineering.

## Figures and Tables

**Table 1:** Sample abbreviation at different RF powers.

Polymer	Percentage of PCL 2b20 to 4b10 Macro Monomer	RF Power	Sample Abbreviation
PDMS	-	30	PDMS0-A
	-	60	PDMS0-B
PCL	1:1	30	PCL1-A
	1:1	60	PCL1-B
	7:3	30	PCL2-A
	7:3	60	PCL2-B

**Table 2:** Elemental composition after CF_4_ treatment (30 W) on PCL and PDMS surfaces. In treatment time column, the “reference” represents 0 s or untreated sample surface.

Sample	Treatment Time (s)	Composition (Atomic %)			
		C1s	O1s	F1s	Si2p
PCL1	reference	74.7	24.4	-	0.9
	10	65.6	16.2	17.7	0.5
	30	67.0	17.4	14.9	0.6
	60	66.3	18.2	14.8	0.7
	180	63.0	16.1	20.5	0.4
PCL2	reference	69.2	25.4	-	5.4
	10	66.0	17.3	15.5	1.1
	30	66.9	17.7	14.5	0.8
	60	65.7	17.9	15.5	0.9
	180	67.2	15.5	16.8	0.4
PDMS0	reference	52.4	21.2	-	26.4
	10	47.3	31.0	-	21.7
	30	49.0	26.8	7.5	16.7
	60	47.8	30.1	1.4	20.7
	180	45.0	28.8	7.8	18.4

**Table 3:** Elemental composition after SF_6_ treatment (30 W) on PCL and PDMS surfaces. In treatment time column, the “reference” represents 0 s or untreated surface.

Sample	Treatment Time (s)	Composition (Atomic %)			
		C1s	O1s	F1s	Si2p
PCL1	reference	50.28	23.28	1.25	25.19
	10	62.54	12.04	24.86	0.56
	30	64.75	11.34	23.82	0.09
	60	64.53	10.99	24.45	0.03
	180	64.16	11.65	24.18	0.01
PCL2	reference	43.6	21.77	0.48	34.15
	10	67.84	14.24	17.29	0.63
	30	67.71	12.11	20.07	0.11
	60	64.08	12.01	23.78	0.13
	180	61.95	10.89	27.14	0.02
PDMS0	reference	46.1	19.46	0.23	34.3
	10	45.52	24.27	2.78	27.43
	30	44.86	24.46	3.45	27.23
	60	43.1	25.93	3.27	27.49
	180	47.97	20.26	3.95	27.82
